# Endocrinological Manifestations of Sanjad-Sakati Syndrome

**DOI:** 10.7759/cureus.8770

**Published:** 2020-06-22

**Authors:** Masharib Bashar, Muhammad Taimur, FNU Amreek, Khalid A Sayeed, Amber Tahir

**Affiliations:** 1 Internal Medicine, Dr. Ruth KM Pfau Civil Hospital, Karachi, PAK; 2 Internal Medicine, Dow University of Health Sciences, Karachi, PAK; 3 Surgery, New York University School of Medicine, New York, USA; 4 Internal Medicine, Liaquat College of Medicine and Dentistry, Darul Sehat Hospital, Karachi, PAK

**Keywords:** sanjad-sakati syndrome, hypoparathyroidism-retardation-dysmorphism syndrome, hypoparathyroidism, pediatric rare diseases, autosomal recessive, endocrinology

## Abstract

Sanjad-Sakati syndrome (SSS), also known as hypoparathyroidism-retardation-dysmorphism (HRD) syndrome, is a very rare genetic disorder with an autosomal recessive mode of inheritance, mostly seen in children of Middle Eastern origin. Hypoparathyroidism remains the most characteristic endocrinological feature of SSS; but not the only one. This review outlines and elucidates other endocrinological manifestations that may be seen with this syndrome.

## Introduction and background

First reported in Saudi Arabia by Sanjad et al. in 1988 [1], Sanjad-Sakati syndrome (SSS), also known as Richardson-Kirk syndrome, has been listed in Online Mendelian Inheritance of Men (OMIM) no. 241410 as hypoparathyroidism-retardation-dysmorphism (HRD) syndrome. In 1991, the mode of inheritance and its configuration was confirmed by the same team of specialists at King Faisal Specialist Hospital and Research Centre, Saudi Arabia [2].

SSS is a rare, autosomal-recessive syndrome, found exclusively in children of Arabian origin, however, case reports from non-Arab countries have also been reported [3]. Its prevalence is not well established, however, the estimated incidence in Saudi Arabia varies from 1:40000 to 1:600000 live births [4].

As indicated in the term HRD, hypoparathyroidism remains the primary endocrinological manifestation. The affected children may present with hypocalcemic tetany, hyperphosphatemia, physical and mental growth retardation, seizures, and craniofacial and orodental deformities, usually during the first few weeks of life. This study aims to explore other endocrinological manifestations that may be seen in this condition as reported in the past, such as hypothyroidism and growth hormone deficiencies. The exact pathophysiology behind these hormonal imbalances and their correlations is yet to be found [2,5].

## Review

Pathophysiology

SSS is caused by a mutation in the tubulin co-factor E (TBCE) gene, the locus for which is located in chromosome 1q42.3. TBCE gene encodes a molecular chaperone that is required for joining of two different subunits, namely, alpha-tubulin and beta-tubulin (heterodimerization) [6,7].

Microtubules are one of the three types of cytoskeletal fibers. These fibers are important for various intracellular processes, for example, cell division, cell motility, and intracellular transport. After the heterodimerization of alpha and beta-tubulins, a quaternary structure is formed. In the absence of a molecular chaperone, these tubulin proteins, interact with other cellular proteins via hydrophobic interactions that prevent them from achieving the quaternary structure required for their biological functions. Since these tubulin proteins are ubiquitously found in all cells of the body, this explains the pleiotropic manifestations of SSS. However, most features affect the central nervous system (CNS) and musculoskeletal systems which are a reflection of high tubulin concentrations in those sites of the body [6].

Courtens et al. (2006) reported a unique variant of SSS not associated with TBCE gene mutation. A de novo micro-duplication on chromosome 4q35 was detected by microarray analysis, with normal TBCE gene on mutation analysis and presence of TBCE protein following immunostaining was observed on lymphoblastoid cell obtained from the patient. Although Courtens et al. concluded that the second gene locus for this disorder seems probable, additional studies could not confirm their finding [8].

Clinical presentation

Newborns with SSS may have prominent craniofacial features like microcephaly, deep-set eyes, beaked nose, depressed nasal bridge, the difficulty of feeding in infancy, long philtrum, thin lips, micrognathia, small hands and feet, teeth anomalies and thick large floppy ear lobes. Prenatal and postnatal severe growth retardation may be appreciated. Occasionally, immune defects secondary to hyposplenism are also seen. Most patients present within the first few weeks of birth with tetany, apnea, seizures, and Chvostek sign (elicited by tapping on the angle of mandible). These signs occur due to hypocalcemia secondary to primary hypoparathyroidism [9,10].

The classical and universal endocrinological feature of SSS remains primary hypoparathyroidism. However, after reviewing the literature, we found it pertinent to discuss the other endocrinological problems reported [11-13].

In one report published in 1996, it was reported that a SSS patient had short stature secondary to a deficiency in growth hormone (GH) secretion, as confirmed by the failure of two formal stimulatory tests. In addition, the lack of testosterone response to human chorionic gonadotrophin (HCG) therapy, reflected primary hypogonadism [11]. A series of case reports published by Hafez et al., also showed that SSS patients had subnormal serum GH levels, and no appreciable rise was observed following arginine and L-3, 4-dihydroxyphenylalanine (L-DOPA) stimulation, however, some rise in level was observed following clonidine stimulation [12]. Most importantly, a marked increase in their heights and weights were observed after treatment with human GH. Hershkovitz et al. found decreased serum insulin-like growth factor 1 levels (IGF-1) in all their patients, and GH deficiency in one patient [13]. This further showed that not only there is hypopituitarism selectively involving somatotrophs in these patients, but perhaps, some degree of GH resistance may also be present, which accounted for low IGF-1 levels [13].

Anteet et al. recommended regular thyroid screening in SSS patients because they found deranged thyroid status in one-third of his cohort of SSS patients. In most of such patients, subclinical autoimmune hypothyroidism (Hashimoto's) was the rule, and several had positive thyroid autoantibodies. They mentioned autoimmune thyroiditis as the cause of thyroid defect in their SSS patient [14].

It is also believed that since the microtubular (MT) defect is the main disease mechanism of SSS, it may affect every cell in the body to some extent. Microtubular-mediated maturation and hypertrophy of chondrocytes at the growth plates are very important for the longitudinal growth of long bones. Hershkovitz et al. suggested that the primary failure of long bone growth plate function might be the reason for severe growth retardation seen in SSS patients [13]. A combination of defects affecting GH, IGF-1 and thyroid hormone secretion coupled with malnutrition caused due to recurrent infections and growth plate defects, may all play the role in the development of short stature in these patients and perhaps, may also impede the brain development for which normal thyroid function is so necessary especially during the first year of life [13].

We believe that as more reports on this rare condition are being published, we will know more about the associations related to this condition. Moreover, the exact mechanism behind these hormonal dysfunctions shall be determined, which will open a wide area of research to identify other genetic possible genetic defects in SSS and the associated hormonal abnormalities. For now, the facts mentioned above are sufficient to prove that a range of endocrinological defects is observed in these patients, and hence, while evaluating these patients, we must keep in mind to holistically approach the endocrinological system.

Assessment and diagnosis

Suspected children must undergo a detailed general physical assessment. Particular regard has to be given towards appreciation of the physical musculoskeletal features like salient facial appearances as mentioned above. The most important issue in such patients is to exclude other conditions, which may present in a similar manner, but whose management and counseling are entirely different, for example, Di-George syndrome, Kenny-Caffey syndrome (KCS) type 2, and familial hypoparathyroidism. The presence of characteristic dysmorphic features rules out familial and X-linked hypoparathyroidism [15].

Complete blood picture (CBC) of all suspected patients must be evaluated. Maintenance of blood components within their normal range, especially the T-cell count, effectively rules out Di-George syndrome. In Di-George syndrome, hypoparathyroidism is combined with thymic aplasia causing defective T-cell-mediated immunity. Cardiac and immunological defects may be absent in certain Di-George cases with incomplete penetrance [15], but the absence of typical facies like cleft-palate may help rule out Di-George Syndrome.

KCS type 2 is a very rare, autosomal dominant disease, characterized by similar episodes of hypocalcemia secondary to hypoparathyroidism as well as typical short stature, like in SSS. However, most KCS type 2 patients have normal intelligence, unlike in SSS, and exhibit macrocephaly secondary to a much wide-spaced fibrous joint between bones of forehead. The anterior fontanelle is large and closes late in KCS type 2 patients. Such patients also have thickened outer cortices and thin medullary cavities (medullary stenosis) affecting several bones in the body. It is pertinent to mention that, the recessive form of KCS (KCS type 1) was actually found to be the same syndrome as SSS by different investigators [16].

Another important investigation in this condition is the measurement of serum electrolytes, in which the most important one is obviously serum calcium and phosphorus. Decreased serum calcium and elevated phosphorus are expected secondary to hypoparathyroidism. Absent or decreased serum parathyroid hormone (PTH), confirms hypoparathyroidism as the cause of hypocalcemic symptoms. Intracranial (particularly basal ganglia calcifications) can be seen on brain imaging by CT/MRI secondary to hyperphosphatemia. In rare cases, partial agenesis of the corpus callosum can also be observed [17].

Treatment

The treatment of patients suffering from SSS is a special challenge for all physicians, especially with controlling high phosphate levels in the blood which leads to generalized calcifications affecting multiple body systems, particularly, basal ganglia calcifications, in which globus pallidus is the most common site to be involved [17,18].

Since hypoparathyroidism is an almost universal complaint in such patients, the primary management includes treating that defect. If such patient presents with symptoms of acute hypocalcemia, the treatment involves intravenous (IV) bolus of 9-15 mg elemental calcium/kg (1 g calcium gluconate = 90 mg elemental calcium), administered over 10-30 mins [19]. Once serum calcium, is within a safe range (>7.5 mg/dL), IV calcium can be stopped. Oral calcium and calcitriol (active Vitamin D), should be started as soon as possible, however, an observation period of 24 hours is recommended to look for rebound hypocalcemia, to ensure the success of oral therapy [20]. Oral calcium is initiated for a total of 100 mg elemental calcium/kg/d divided four times daily. Once serum calcium concentrations range from 8-9 mg/dL, the calcium dose is weaned to the minimum dose necessary to maintain low-normal serum calcium. Synthetic parathyroid hormone therapy has so far not been approved for children suffering from chronic hypoparathyroidism [19]. Renal stones have to be managed either surgically or medically, depending on its size and duration.

Regarding other endocrinological dysfunctions, GH deficiency has been shown to be usually unresponsive to GH therapy in SSS patients [21]. Hypothyroidism has to be treated as for any other cause with levothyroxine, with the treatment tailored according to the current thyroid status of the patient.

Since SSS is an incurable disease, and treatment entirely focuses on the management of individually presented conditions. It is recommended that genetic counseling must be given to the affected parents. Prevention of this condition will be made possible in the future, through pre-implantation genetic diagnosis and carrier detection [9]. 

Prognosis

SSS is an incurable condition, and management is mostly limited to palliation. Functional hyposplenism leads to recurrent infections, especially in the respiratory system, and this is the cause of early death in most children, however, rare cases have survived up to the age of 18 years. An interesting, yet unexplained complication in older children that have been seen in few cases is severe constipation with gut dilatation. Complications related to disordered calcium-phosphate metabolisms, such as intracranial calcification and corneal opacification, are amenable to adequate replacement therapy, however, most of the skeletal defects are irreversible. Growth defects have shown resistance to treatment with GH therapy, as mentioned above. By and large, SSS is a chronic, progressive disease, for which palliation is the mainstay for management, but ultimately, recurrent infections limit the lifespan of most patients to below 18 years of age [10].

## Conclusions

This study has shown that apart from the parathyroid gland, other endocrine organs are equally affected in SSS. Hopefully, as we see more case reports and articles related to this rare condition, further endocrinological manifestations of this disease will be found. Hence, it is the responsibility of all clinicians that whenever encountering patients suffering from SSS, they should conduct a detailed endocrine workup even if symptoms related to derangements of those organs are absent.

## References

[1] Sanjad S, Sakati N, Abu-Osba Y (1988). Congenital hypoparathyroidism with dysmorphic features: a new syndrome. Pediatr Res.

[2] Sanjad SA, Sakati NA, Abu-Osba YK, Kaddoura R, Milner RD (1991). A new syndrome of congenital hypoparathyroidism, severe growth failure, and dysmorphic features. Arch Dis Child.

[3] Sen C, Pal S, Sengupta P, Pal A, Ganguly J, Das C, Basu D (2016). Sanjad-Sakati syndrome: beyond the Middle-East. Indian J Cereb Palsy.

[4] Teebi AS (2000). Hypoparathyroidism, retarded growth and development, and dysmorphism or Sanjad-Sakati syndrome: an Arab disease reminiscent of Kenny-Caffey syndrome. J Med Genet.

[5] El Batawi HY (2013). Sanjad-sakati syndrome dental management: a case report. Case Rep Dent.

[6] Padidela R, Kelberman D, Press M, Al-Khawari M, Hindmarsh PC, Dattani MT (2009). Mutation in the TBCE gene is associated with hypoparathyroidism-retardation-dysmorphism syndrome featuring pituitary hormone deficiencies and hypoplasia of the anterior pituitary and the corpus callosum. J Clin Endocrinol Metab.

[7] Parvari R, Hershkovitz E, Kanis A, Gorodischer R, Shalitin S, Sheffield VC, Carmi R (1998). Homozygosity and linkage-disequilibrium mapping of the syndrome of congenital hypoparathyroidism, growth and mental retardation, and dysmorphism to a 1-cM interval on chromosome 1q42-43. Am J Hum Genet.

[8] Courtens W, Wuyts W, Poot M (2006). Hypoparathyroidism-retardation-dysmorphism syndrome in a girl: a new variant not caused by a TBCE mutation--clinical report and review. Am J Med Genet A.

[9] Cader SHA, Shah FA, Nair SKGR (2016). Otolaryngologic manifestations of Sanjad Sakati syndrome-a case report. Otolaryngol Online J.

[10] Arabi WA, Basheer AA, Abdullah MA (2011). Sanjad-Sakati syndrome in Sudanese children. Sudan J Paediatr.

[11] Soliman AT, Darwish A, AlSalmi I, Asfour M (1996). Defective growth hormone secretion and hypogonadism in the new syndrome of congenital hypoparathyroidism, growth failure and dysmorphic features. Indian J Pediatr.

[12] Hafez M, Anwar GM, Ibrahim A, Musa N (2017). Sanjad Sakati syndrome: case reports from Egypt. Egypt Pediatr Assoc Gaz.

[13] Hershkovitz E, Rozin I, Limony Y, Golan H, Hadad N, Gorodischer R, Levy R (2007). Hypoparathyroidism, retardation, and dysmorphism syndrome: impaired early growth and increased susceptibility to severe infections due to hyposplenism and impaired polymorphonuclear cell functions. Pediatr Res.

[14] Anteet AM, Al Issa ST, Al-Ali AO, Al-Otaibi HM, Mohamed S, Babiker A, Al-Jurayyan NA (2016). Autoimmune thyroiditis associated with Sanjad-Sakati syndrome: a call for regular thyroid screening. Sudan J Paediatr.

[15] Swillen A, Vogels A, Devriendt K, Fryns JP (2000). Chromosome 22q11 deletion syndrome: update and review of the clinical features, cognitive-behavioral spectrum, and psychiatric complications. Am J Med Genet.

[16] (2020). Kenny-Caffey syndrome - NORD (National Organization for Rare Disorders). https://rarediseases.org/rare-diseases/kenny.

[17] Alghasab N, Janati AB, Khan A (2012). Partial agenesis of corpus callosum in Sanjad-Sakati syndrome (p-ACC). Can J Neurol Sci.

[18] Albaramki J, Akl K, Al-Muhtaseb A, Al-Shboul M, Mahmoud T, El-Khateeb M, Hamamy H (2012). Sanjad Sakati syndrome: a case series from Jordan. East Mediterr Health J.

[19] (2020). Pediatric hypoparathyroidism medication: calcium supplements, vitamin D supplements. https://emedicine.medscape.com/article/922204-medication.

[20] (2020). Pediatric hypoparathyroidism treatment & management: medical care, diet. https://emedicine.medscape.com/article/922204-treatment.

[21] Rafique B, Al-Yaarubi S (2010). Sanjad-Sakati syndrome in Omani children. Oman Med J.

